# Artemin and Its Emerging Role in Pathogenesis of Systemic Tumors Besides Pancreatic Cancers

**DOI:** 10.3389/fonc.2012.00092

**Published:** 2012-08-08

**Authors:** Shailendra Kapoor

**Affiliations:** ^1^University of Illinois at ChicagoChicago, IL, USA

TO THE EDITOR: The recent article by Demir et al. (2012) in your esteemed journal provided for highly stimulating and interesting reading. Interestingly, over the past few years artemin has been identified as a significant player in the enhancement of oncogenicity of various other tumors besides pancreatic cancers.

For instance, artemin enhances transcription of bcl2 – leading to it's up regulation and thereby augments tumor growth in human non-small cell lung carcinomas (Tang et al., 2010). Similarly, in estrogen receptor negative breast carcinomas, artemin shows synergization with TWIST1 and thereby accentuates the metastatic potential of the primary breast tumor (Banerjee et al., 2011). As a result, a poor clinical outcome is associated with this combination of artemin and TWIST1. Attenuated artemin expression is seen as a result of tamoxifen administration (Kang et al., 2010). Interestingly, the sensitivity to tamoxifen of tamoxifen resistant mammary tissue is accentuated following antibody mediated inhibition of artemin.

Increased expression of artemin is also seen in esophageal carcinomas. Interestingly transfection with a mir-223 vector decreases expression of artemin and thereby suppresses tumor growth in esophageal carcinomas (Li et al., 2011). Similarly, artemin augments the expression of AKT1 and thereby accentuates the invasive potential of endometrial carcinomas (Pandey et al., 2010). The invasive potential of endometrial cancer tissue is significantly abrogated following antibody mediated inhibition of artemin.

The above examples clearly illustrate the significant enhancement of oncogenicity secondary to artemin in tumors ranging from lung carcinomas to endometrial carcinomas. There is a clear and urgent need to identify inhibitors of artemin function in order to improve the prognosis in these tumors.
